# Taking social relationships seriously: Lessons learned from the informed consent practices of a vaccine trial on the Kenyan Coast

**DOI:** 10.1016/j.socscimed.2008.02.003

**Published:** 2008-09

**Authors:** Caroline Gikonyo, Philip Bejon, Vicki Marsh, Sassy Molyneux

**Affiliations:** a KEMRI-Wellcome Trust Research Programme, P.O. Box 230, Kilifi, Kenya; b Centre for Clinical Vaccinology and Tropical Medicine, University of Oxford, Churchill Hospital, Oxford, UK

**Keywords:** Informed consent, Vaccine trials, Rumours, Clinical research, Testing understanding, Developing countries, Kenya

## Abstract

Individual informed consent is a key ethical obligation for clinical studies, but empirical studies show that key requirements are often not met. Common recommendations to strengthen consent in low income settings include seeking permission from community members through existing structures before approaching individuals, considering informed consent as a process rather than a single event, and assessing participant understanding using questionnaires. In this paper, we report on a qualitative study exploring community understanding and perceptions of a malaria vaccine trial (MVT) conducted in a rural setting on the Kenyan Coast. The MVT incorporated all of the above recommendations into its information-giving processes. The findings support the importance of community level information-giving and of giving information on several different occasions before seeking final individual consent. However, an emerging issue was that inter-personal interactions and relationships between researchers and community members, and within the community, play a critical role in participants' perceptions of a study, their decisions to consent or withdraw, and their advice to researchers on study practicalities and information to feedback at the end of the trial. These relationships are based on and continually tested by information-giving processes, and by context specific concerns and interests that can be difficult to predict and are well beyond the timescale and reach of single research activities. On the basis of these findings, we suggest that the current move towards increasingly ambitious and stringent formal standards for information-giving to individuals be counter-balanced with greater attention to the diverse social relationships that are essential to the successful application of these procedures. This may be assisted by emphasising respecting communities as well as persons, and by recognising that current guidelines and regulations may be an inadequate response to the complex, often unpredictable and ever shifting ethical dilemmas facing research teams working ‘in the field’.

## Informed consent in international biomedical research settings

Individual informed consent is a key ethical obligation for clinical studies, but empirical studies show that key requirements are often not met. Problems identified in high income settings include: difficulty in getting participants to fully understand and accept the facts and their implications; overcoming patient fear of reproach if they do not conform to doctors' requests; reluctance of doctors and participants to acknowledge uncertainties in modern medicine; a difficulty in transcending patients' trust in medicine and their doctors; over-emphasis on the legal as opposed to moral aspects of consent; and insensitivity and authoritative handling of vulnerable people by doctors). These challenges can be compounded in international research environments by relatively big differences between investigators and community members in levels of formal education, in access to good quality biomedical services, and in values, priorities and understandings of health and illness (Benatar, 1994; Doumbo, 2005; Emanuel, Wendler, Killen, & Grady, 2004; Leach & Fairhead, 2005; Qiu, 1993). Underlying these differences are global and local inequities in access to information and resources (Benatar, 2002).

Most guidelines continue to recommend informed consent in low income settings; the majority of ethical committees retain it as a key requirement (CIOMS, 2002; NCOB, 2002; WMA), and it is now a matter of law in the European Union (EU, 2006). Recommended adaptations include preceding individual consent with community level consultation and permission, typically through existing structures of authority such as chiefs, community and religious leaders, and schools. Where the majority of potential participants cannot read or write, alternative methods of documenting the individual informed consent process such as using audio or video tape might also be considered. Further suggestions for improving informed consent, many of which are not unique to low income settings, are (Allmark, Mason, Gill, & Megone, 2003; Edwards, Lilford, Thornton, & Hewison, 1998; Fitzgerald, Marotte, Verdier, Johnson, & Pape, 2002; Kass, Maman, & Atkinson, 2005; Marshall, 2006; Mitchell, Nakamanya, Kamali, & Whitworth, 2002; Molyneux, Peshu, & Marsh, 2004; Siminoff, 2003; Smyth & Weindling, 1999):

• Allowing for shorter, more simplified wording in consent forms, as much of the detail currently requested in consent forms is designed to protect researchers and their institutions rather than research participants;
• Considering informed consent as a process rather than a one-off exercise, and as part of a wider community engagement process;
• Incorporating visual aids and simple verbal tests, or quizzes, into informed consent processes;
• Carrying out preliminary or concurrent social science research to check understanding and identify local issues and concerns to consider in information-giving activities;
• Careful participatory training of fieldworkers (FWs) in communication skills and ethics; and
• Considering ‘assent’ only on admission, or developing a ‘step-like’ or ‘continuous’ consent process for emergency situations.

In this paper, we report on community understanding of and perceptions surrounding the informed consent processes of a malaria vaccine trial (MVT) conducted in a rural setting on the Kenyan Coast. The MVT is one study carried out by the KEMRI/Wellcome Trust Research Programme. This is a large multi-disciplinary research programme with two closely linked ‘Units’ – Nairobi and Kilifi. The research programme and how it is perceived by the local resident population are described in detail elsewhere (Marsh, Kamuya, Gikonyo, Rowa, & Molyneux, 2008; Molyneux et al., 2004; Molyneux, Peshu, & Marsh, 2005; Molyneux, Wassenaar, Peshu, & Marsh, 2005). Included in previous papers are information on understanding of research details of three studies, which appeared low, and on rumours and concerns including surrounding blood volumes and use, and the need for clearer employment policies.

The MVT was selected for this study because we have not previously looked at perceptions and understanding of a vaccine trial, because the information-giving practices included many of the recommendations made locally and in the literature, and because FWs from the study villages were employed to live in the villages over the study period. The more common practice in our institution at the time was to employ staff from across the district to work on a series of studies; usually resulting in their being resident in Kilifi town and making daily visits from the research centre to study households. For the MVT study, two senior FWs, with prior experience of clinical trials, were based in Kilifi town, to supervise 10 FWs resident in the study area.

The aims of the community perceptions study presented in this paper were to:

1. document participants' understanding and concerns at the point of recruitment into the MVT;
2. explore community members' views about the MVT, the amount of information given, and the consent processes followed;
3. gather community members' expectations and inputs into information for feedback and methods to use at the end of the MVT; and
4. gather community member and staff perceptions on the pros and cons of having field-based interviewers working on community-based studies.

In the following sections we describe the MVT in more detail, followed by the methods and key findings corresponding to the above objectives. The study was not designed to explore social relationships, but their importance throughout the findings is clear, and returned to in the discussion as an emerging issue.

## The malaria vaccine trial – overview and information-giving procedures

The MVT centred on two candidate vaccines FP9 ME-TRAP and MVA ME-TRAP, used in sequence. The trial aimed to document the vaccines' safety, immunogenicity and efficacy against febrile malaria in 400 children using a double blind randomised controlled trial (with rabies vaccine as the control). The study procedures are summarised in Box 1 (see also Bejon et al., 2006). Parents were encouraged to contact field staff based in the study villages in the case of any illness in study participants. The FW could then communicate with the PI by mobile phone in the case of an emergency, or directly with the dispensary, to ensure the child received treatment. This was free of charge for the one year study period. The MVT used a multi-step informed consent process, summarised in Box 2. Each step was used to respond to issues raised at the previous step. All preliminary information-giving activities were held within 3 months, and were supported by distributing information sheets in the local languages. The entire process, including consent forms and information sheets, had been reviewed in advance by a consent committee at the unit, as well as the national and international Institutional Review Boards.

## Methods

Participant understanding and perceptions of MVT were explored through two main activities, both of which were conducted by interviewers independent of the trial team (members of the institution's Social and Behavioural Research Group, or ‘SBR’).

### Exit interviews/quiz

A quiz passed by the local and national ethics review committees had initially been incorporated into step four of the above information-giving process. For the purposes of this study, the quiz was implemented as an exit interview with two sections: (a) open-ended questions seeking basic information on where the person learned about the MVT, what they liked/did not like about the study and reasons for joining; and (b) open-ended questions checking the understanding of key issues that had been dealt with during the informed consent process. Exit interviews were conducted with 189 caretakers after the consenting and screening processes. The quiz was administered at the last possible moment to avoid disrupting the screening process. Issues that were raised by participants were referred to trial staff to ensure that participants did not leave with prominent concerns.

### Individual interviews and focus group discussions

Individual interviews were held with the PI and senior FWs working on the MVT, but based in Kilifi (*n* = 3). Group discussions were held with the FWs based in the study area (*n* = 2) and with community members who consented to be in the study (*n* = 9). These discussions took place on average 5 months after the initial consenting process and almost halfway through the trial. All community group participants were female, and their children had received all three vaccinations. Focus Group Discussions (FGDs) explored issues around objectives 2–4 above. The FGDs were held until a ‘point of saturation’ was reached and no new information was being collected (de Negri & Thomas, 2003). The discussions were conducted in the local dialect, tape-recorded, transcribed and translated into English.

Exit interview answers were grouped and entered in Foxpro and frequencies produced using SPSS version 11.5. A detailed thematic framework was developed and applied to the qualitative data by the first author and checked by the last author and an independent colleague. Quotations presented in this paper have been selected to illustrate typical or particularly illustrative comments made under the main themes that emerged. In a separate paper, we discuss community reactions to the exit interview/quiz itself in more detail (Molyneux, Gikonyo, Marsh, & Bejon, 2007).

## Findings

### Community understanding of the MVT and reasons for joining and staying

Of the 189 exit interviews, 91% were with the participant's mother, 2% with fathers and 7% with other caregivers, primarily aunts and grandmothers. These figures reflect the general picture of those bringing children to the dispensary for the screening and vaccine visits. Of quiz respondents, 40% said the mother decided on the child's involvement, 19% said both parents and 18% the father alone. In some cases, it was reported that the father simply informed the mother to take the child to the dispensary and enrol him or her, without informing the mother why or what the project was about.

The proportion of participants with correct responses ranged from 29 to 85%, depending on the question (Table 1). The proportion reporting they do not know ranged from 14 to 45%. This suggests very variable levels of knowledge for different and even highly related key sets of information, and some major gaps in understanding. However, our interview questions may not have fully captured participant understanding; because of the tool itself, or the way and point at which it was administered. In FGDs, for example, comments suggested that many parents did understand that the study was a malaria vaccine trial. Hints of this understanding were sometimes found when discussing other issues. For example, as one mother said when discussing study benefits:

About one month ago my child experienced fever and vomited yellow and her stool was yellow, so I don't know if she was given the rabies or the malaria vaccine because she still has problems… and that vaccine is still being tested to see if it can protect against malaria or not (KI; P12; page 12).

The trial was generally described as some form of assistance; as a project aimed at ensuring good health in children through prevention, treatment and check-ups. This was true also for those who apparently understood the research aims of the trial. Linked to this finding, the main reasons for joining and staying in the study were reported by most to be the individual benefits associated with the study such as free treatment, transport to and from Kilifi District Hospital in the case of any illness, and access to the PI (who is medically qualified). These individual benefits were repeatedly highlighted over any altruistic interest in contributing to the global pool of knowledge on malaria prevention:

What attracted us [was that] we knew our children will receive treatment for a whole year in every disease they suffer. If you have a problem and visit the people concerned, a call is made to the … [PI] he brings a vehicle and [the sick person] is carried away [to hospital]. In fact it's something we should be happy about because nobody can bring you a vehicle that easily… (G1; P11; page 11).

In some cases, the decision to join the study had less to do with the any of the study details (scientific or practical) than with previous encounters with, and confidence in, the research centre:

I had another baby who suffered epilepsy and some white people were registering epileptic children. A time came when they invited me 3 times to take her there giving me fares and other free offers… it was ages ago… and people in my home said ‘all those free offers?, you'd better not go!’. But I went… and the baby took drugs, her condition got better and now she's OK. So now, when this KEMRI activity about malaria came, I saw no problem. I hurriedly went and underwent their processes (KI; P7; page 16).

### Concerns with the study

Of the quiz respondents, 22% expressed at least one concern about the vaccine trial or KEMRI. This was not on direct questioning, but emerged in the process of administering the quiz. Most concerns (44%) were related to the vaccine (fear of side-effects, that it was a trial vaccine, that they were not being told which vaccine their child was getting, and that the vaccine was not being given to all children). Other concerns were about the blood samples (21%), why photographs were being taken (16%), rumours circulating in the community (described below), whether there was to be HIV testing, and personal concerns (9% each). Most personal concerns were from mothers who were worried about their husbands' reaction when they found out that the mother had enrolled the child/ren without the father's consent. Most of these mothers reported wondering what would happen if the father decided to withdraw the child.

These concerns were mirrored in the FGDs. Most were voiced as concerns of the community, and linked to rumours about KEMRI being a ‘devil worshipping organisation’. Reported bases for these rumours are summarised in Box 3, with several often mentioned together as follows:

P8: It is said that we joined KEMRI and photographs were taken, blood was removed and both will be taken there [to KEMRI]… later they will cut the photo up and the child will start fitting

P3: Yes, the child will fit [*i.e., have a seizure*] [laughter]… and die… so KEMRI are devil worshippers (M2; page 3).

Some participants said that *other people* became particularly fearful when they heard or read about the side-effects detailed in the information sheet. Such rumours and concerns were reported as the main reasons behind non-participation and drop-outs, and to flare up when rumours appeared to be being proven true:

At the time [of the first vaccine being administered]….. One of us went in while her child was asleep and got the injection. [The child] continued to sleep even after coming out of the room. A mother among us said, ‘Aaaahh the child has fainted!’ That is when many started to …withdraw. It was the first group…The first group created all these problems… (M1; P2; page 20).

When asked about *their own* concerns now, it appeared that there was some concern that the devil worshipping rumours may yet be proven true:

P12: they [non-participants] are out to worry us…

P4: It's a conflict between those who attended and those who didn't, so it's upon us to educate them so that they don't convince the ones participating to withdraw…

P8: When they see us boarding the free vehicles they shout ‘a lazy person takes advantage of any chance’ (M2; pages 4–8).

…the [vaccine] for malaria is still new in our place that's why they are doing it [the trial] using our children. And a lot of nonsense has been going round. We are fighting to cross over [to truly believing that all of the rumours are nonsense] but after [the trial] you should think about us because we are in the middle of water (laughter) [i.e., the ones taking the risks] we don't know whether we'll drown or what … we are in the middle of the sea. (K2; P2; page 24)

The above comments suggest that many rumours were being spread by non-participants because of intra-community conflicts and jealousies centring on the trial. By the time of the discussions, participants appeared to have the upper hand: they were enjoying the free treatment provided as part of the trial and none of the rumours had yet been proven true. In all groups, there was mention of non-participants regretting their decision and participants telling them that they were too late. Others talked of non-participants coming to borrow drugs for their sick children, in some cases being told off by participants. Nevertheless, there was often some indication of continued concern in what was often described as their journey with their research team: “…even though we have worries, we cannot drop-out now because we have already joined…”; or “in a traffic accident, the driver dies with the passengers”. Everyone expressed hope that at the end of the study, their concerns would be alleviated and their decision to remain participants appreciated by the study team and envied by non-participants.

### The information-giving process for the study

During the quiz, 35% of the caregivers reported hearing about the vaccine trial during community meetings, 34% from vaccine trial field assistants visiting their homesteads, 21% from relatives and neighbours, and 6% from the local dispensary.^1^ Most (58%) said that they learned most when they came for the screening and a fifth that information from all sources was equally important.

In FGDs, there was strong support for each of the stages followed in the information-giving process. The involvement of the local chief, elders and dispensary committee at the outset was described as essential for an outside group or individual entering the local community:

P7: [It was] good because he passed through the government; we saw him first with the chief. That made us feel peaceful because he was with the chief, a village elder and our hearts were clean because we know if any bad thing befalls us, we'll first get hold of the chief or the village elder to solve that problem (K1, P3/7, page 19).

Information-giving at the individual household level was described as offering greater opportunity for discussion and ensuring husbands were included in information and decision-making processes:

P3: …those [public] meetings were attended mostly by women who are usually ‘yes’ people and so things sometimes go wrong in their hands. But in the homestead you can find the husband… if he comes to listen he'll have questions and after all [the questions] have been answered, that's when a decision will be made…

P10: …I went to the meetings and was visited at home. That gave me the motivation to go to the dispensary and find out even more and to decide there if I like it I'll enrol my child, if not I won't (K1; page 23).

Specifically and regularly mentioned was the PI's presence, his efforts to speak the local languages, and their ability to question him directly about the study. Overall, parents reported that the community had been well informed about all aspects of the study before it began, and that people joined of their own free will knowing that they could withdraw whenever they wished. Of particular note was apparent appreciation of having been informed of ‘the good and bad sides’ of the study before joining (i.e., the benefits vs side-effects). In general, there were no suggestions about what more could have been done to alleviate rumours beyond proving them wrong, because ‘one individual cannot clean another person's heart’.

General disadvantages raised in the FGDs regarding the informed consent process were that the side-effects and the voluntary nature of the participation were over-emphasised, and the signing of the consent form itself. These emphases were viewed by some as the project or KEMRI protecting themselves should anything happen to participants (e.g., KEMRI not being blamed if there were severe side-effects):

We were explained to and understood that it wasn't a must. It was a personal decision. You agree then the child goes in for screening. We were asked at least three times. Just like when you see someone preaching on the roadside you may think he is mad but he's not, you just think s/he is because you don't know what is in the bible. For us who are … [in the study] we understand [the risks and benefits] (laughs). [We understand that it's] just like in a vehicle accident where your friends might die and you survive, so we are in because we made that decision. (J2; P13; page 8)

Then you are told to put your signature and after signing your child will be vaccinated… now you have put a noose around your neck… you cannot free yourself (G1; P2; page 10).

In FGDs with fieldworkers, one argued that on the MVT consent form the ‘…[commitment from the PI] did not have the same weight as the [parent's] one that says “I, (so and so), being the mother of this child, have given out my child to be researched on”. The view expressed here, by someone who administers the forms, is that the signing of the consent form indicates a binding, unequal commitment, despite wording on the form itself to the contrary.

### Having locally employed and based FWs working on the vaccine trial

Having locally employed and based FWs was seen as positive both for the project and for the community for several reasons.

• They knew the area very well, and how to talk to, reassure and explain the study to people; helping them understand, and making recruitment faster and easier.
• The close proximity of the FWs, constant surveillance by the team and ease of communication over the follow-up period were important benefits to participants; and
• It was satisfying to see that KEMRI had created employment in the area.

FWs were aware of their importance for the project. As one put it, “we could have spoilt or made the mradi [project] because people in my village were asking questions”. They also had to respond to unexpected issues and questions as they arose, in some cases on the basis of little training. Some responses may have been more appropriate than others. Compare for instance the ideas on how to respond to community concerns about devil worship (Box 4).

There were also problems associated with FWs being part of the community. For example, rumours about relationships between mothers and FWs were mentioned:

P8: The way [the FWs] are free with us; they can visit in the morning or evening to check on the kid. [So non-participants] have now started spreading rumours that they are not only KEMRI but our boyfriends…

P7: …[they say] ‘Is he the only doctor in this area?’ … and that thing of sending him children even at night for treatment they see that as our excuse to visit him: ‘Where was she going at that hour? Going to her boyfriend’… (Laughter) (M2; page 13/14).

These comments hint at the power and status of fieldworkers in forming the link between local communities and much needed health care, the intra-community jealousies and tensions that can result from those who are included and excluded from these research-related benefits, including employment itself, and the difficulties FWs face in their daily interactions at the interface.

### Expectations regarding information to feedback at the end of the study

During the FGDs, three sets of expectations emerged regarding informational feedback.

The first, often referred to as ‘jambo la kwanza’ (the first thing) was to inform the participants about whether the vaccine was found to be working or not, and which children received which vaccine (rabies or malaria). The second was results of all tests done over the follow-up period on individual participants, information on whether children had received the rabies or malaria vaccine, and a report on the child's health status since receiving the vaccine. The need for simple answers, and the desire for a positive finding, was clear. In all groups, it was noted that should the findings be successful, it would reduce worries and concerns about the vaccine, and about the project and KEMRI in general and reduce the teasing and rumours from non-participants. In all groups, there were also references to negative feelings or reactions if the study was to fail, especially if any rumours regarding the dangers of the study were proven true:

where there is a trial, there is either success or failure…so for example if it fails, we all will be in agony…True. We'll start complaining, directing our complaints to [the PI] (K1; various; page 43).

In most groups, there were requests for information in large meetings rather than to individuals or small groups, ‘otherwise there'll be misunderstandings with people saying different results were given to different people’. There was also a call for separate feedback meetings for the general community and for the participants' parents. In some groups, it was expressed strongly that non-participants should not get any feedback from the team but should wait and get it second-hand from participants. One participant emphasised that non-participants should get the finding ‘that makes them feel bad’. The importance of inter-personal interaction as part of feedback was stressed:

because these information sheets you are giving out can either be ignored or considered as mere paper to hide the truth from us… someone can also read from the sheet and misunderstand it completely (M2; page 34/35).

Third, all groups mentioned being concerned about participants being forgotten once the study is over and requested some form of recognition from the PI or KEMRI for their role and the risks and inconveniences they have gone through: recognition ranging from a simple public thank you and acknowledgement, to being given something (‘so the PI does not benefit alone’), to major demands (e.g., building a dispensary near them). Other requests included having a KEMRI employed clinician at the local dispensary to cater specifically for those who participated in the study, field assistants being given enough drugs to last at least one year, and further studies in the area.

“…but he should not succeed and go while we've been together with him… Now, we should be given [presents] openly such that those that did not consent for the study should feel bad …” (K1; various; page 39).

There were also suggestions for KEMRI to find a way of having the vaccine put in the KEPI (Kenya Expanded Programme on Immunisation) schedule and information on whether participants could participate in another trial in the future. Some wondered whether they would be forced to pay for the vaccine once it was made available to all and recommended that there be a way of participants receiving free vaccinations.

## Discussion

### Information-giving processes and understanding

Measuring understanding is far from straightforward. The proportion of respondents giving correct responses differed even for highly related questions in our exit interviews, and comments made in FGDs suggested far greater overall understanding of key study details. The difficulty of measuring understanding, and in particular, of distinguishing between recognition, recall and comprehension, is well recognised. Lindegger et al. (2006), for example, found differences in levels of understanding among the same individuals using four different methods of assessment. They suggested these differences were linked to both under- and over-reporting in response to different types of assessment, and to the methods accessing qualitatively different aspects of the ‘construct called ‘understanding’’ (p 565).

In our study, we believe on the basis of FGDs that respondents' concern about the information being sought, and the relative formality of the process, may have led to an increased number of ‘do not knows’ for some exit interview questions. For example, if parents are being asked (in what was described as a rather intimidating individual ‘exam’ setting), what an activity is about, and are concerned about whether or not they have been told the truth, they may answer ‘don't know’ in the hope of being given new information to confirm or ease any doubts they have (i.e., cross-checking the trial staff). Furthermore, being asked the questions by a similar yet separate group may itself raise concerns about the activity, particularly in a context of rumours circulating in the community, and with the relative novelty of the procedures and social relationships involved in being in a trial. Our findings may also be related to inadequate probing by fieldworkers to open-ended responses, and possibly our posing questions to mothers bringing their children to the dispensary rather than to other household members, particularly husbands and household heads (see also below). These methodological issues and their implications are described in greater detail elsewhere (Molyneux et al., 2007).

The above complexities and limitations aside, participants clearly felt adequately informed about key aspects of the vaccine trial, and overall, understanding of the study appeared greater than we have previously observed (Molyneux et al., 2004). This may be attributed to several factors including (1) the support of experienced local staff in carefully designing informed consent forms, and in the broader information-giving processes; (2) the continuous presence of fieldworkers and the PI in the community, facilitating the building up of rapport between research staff and community members, and quick awareness of and response to emerging issues; (3) the nature of the MVT trial itself, in that it involves well people away from a clinical setting, and a tangible experimental design rather than a more abstract basic science question; and (4) because we explored understanding relatively soon after the intensive information campaigns and administration of the vaccines. There were some informational gaps or misinterpretations, for example, what exactly would happen to the blood, why individual results were not being given for every blood test taken over the one-year period, which vaccine was being tested, and several more unusual concerns such as whether children given the rabies vaccine would later bark. However, many of these sets of information would not generally be included in research information sheets, could not easily be predicted in advance, and were responded to once raised.

Of note are two issues. Firstly, levels of perceived risk as illustrated in rumours and concerns were often far greater and more dramatic than the biomedical risks outlined in consent forms. Information-giving procedures can both contribute to and ease these rumours and concerns, as described in greater detail below. However, the perceived benefits, including free follow-up and treatment of study participants for one year, were also perceived to be substantial. This is not surprising given the significant costs that illnesses present to households (Chuma, Thiede, & Molyneux, 2006). Secondly, and mainly in response to these issues, the main reason people agreed to the study was the very real health benefits that participants and their families would receive, rather than the hope that the vaccine would work. This is common in settings where research activities can come to be considered one of the range of treatment options for families, each of which has its advantages and disadvantages (Leach & Fairhead, 2005).

Study participants were generally supportive of all of the study information-giving procedures. While there may be rather ambivalent views in the community about the position of the chief and community leaders, the recognition and acceptance of the local administration and elders, typically male, is clearly perceived to be essential to the legitimacy of a study before taking any further steps (Molyneux, Wassenaar, et al., 2005), supporting the inclusion of these structures in information-giving. Regarding public community meetings, these are an accepted and widely practised step to entering local communities for all organisations on the Coast, and were considered by all to be an important channel for exchanging information. Discussing the risks and benefits in detail in these forums risks increasing the spread of concerns, rumours and raised expectations in the community, but also enables these issues to be openly discussed and resolved. We believe that the amount of public information-giving required and its impact will depend on the specific study and context, and will be of interest to explore in future studies.

Visits to households are another important step in helping ensure that complex intra-household decision-making dynamics are respected. As we have described elsewhere (Molyneux, Murira, Masha, & Snow, 2002), males and elders generally have the stated judicial authority for economic and health matters in Mijikenda (the dominant ethnic group on the Kenyan Coast) households, while women are almost entirely responsible for supportive care. The extent to which males and elders exert their influence appears to depend on type of household (for example, rural or urban, nuclear or extended, interactions between households); the woman's age, education and income earning role; the specific relationship she has with her husband; what is being decided upon; and who is available or can be made available at the time a decision is needed. Overall, the more ‘risky’ a situation, the more important it is to have the household head, and other elders who are not necessarily the primary caretakers, involved. Given the experimental nature of the MVT, and the unfamiliar procedures and relationships involved, enabling potential participants to consider the study information and implications with other household members was crucial. Our exit interview findings on understanding may have been influenced by those being interviewed not being the main decision-makers around the child's participation, nor the most knowledgeable within the household about the trial. We cannot confirm this given our study design and the difficulty in measuring understanding (described above).

### Rumours, relationships and trust

While there was an overall appreciation of the study details and the information-giving procedures followed, it is clear that participants receive (mis)information about the study and the research institution from other sources too. This information can conflict with or exaggerate study team information, and is often described as ‘rumours’. Examples circulating within the community at the time of our FGDs were around the levels and types of risks related to the blood-taking, vaccines, and other procedures such as photographs and measurement of children in wards. The most prominent were rumours that children would die at the point of being vaccinated, over the course of the study, or even long after the completion of the trial, as a result of witchcraft. Similar rumours have been repeatedly reported across Africa, and should be taken seriously both as a real concern, and as an expression of uncertainty, tension and ethical comment in field settings (Geissler & Pool, 2006). Their strength for communities lies in the difficulty of confronting them by simply re-stating the scientific facts or supplementing explanations with demonstrations. There were strong feelings in our study that rumours were being fuelled and spread by non-participants, and that they may have been inadvertently supported by fieldworkers' own explanations and responses to unexpected questions. What information people chose to believe about the study, associated rumours, perceptions of risks and benefits, and decisions regarding whether or not to join the study, are related to pragmatic interests in treatment and to trust. Trust is a relational notion, describing a voluntary relationship between two or more people (inter-personal trust) or between a person and an institution (institutional trust) (Gilson, 2003). For some individuals trust in the vaccine trial and in the information given out by trial staff was automatically transferred from previous positive interactions with the institution or its staff. Similarly, some of the concerns and rumours were less to do with the vaccine trial information, than to a general distrust in KEMRI and outsiders.

It appears for many that the vaccine trial information-giving process was important not so much for ensuring understanding of study details as for following local rules, helping clear possible misconceptions and fears, and for developing relationships and exhibiting behaviours such as truthfulness, concern, and fairness, that are known to be supportive of trust building (Gilson, 2003; Mechanic & Meyer, 2000). Incorporating into the study team both external technical competence (the PI) and internal inter-personal competence (locally known and based field assistants who are ‘our family’, and ‘who understand us and we can approach’) appeared to be critical. In some cases, the levels of trust in and expectations of the vaccine trial team that were reached were well beyond the aims of the PI. These findings indicate that care is required not to exploit trust once this is established (Molyneux, Wassenaar, et al., 2005). The findings also support the work of others (Elbourne, Snowdon, & Garcia, 1997; Kuczewski & Marshall, 2002) who argue the inadequacy of traditional models of informed consent based on subjects or their proxies making sound, competent, thoughtful and rational choices on the basis of the information given. Some level of fragility in the trust that is built, and an element of enduring scepticism, is essential for the proper functioning of informed consent processes. In our setting this balance was illustrated in the reportedly heavy reliance on observation and experience to decide what information was the truth (‘seeing is believing’), and by apparently sudden concerns and changing of minds by whole groups of potential participants if activities or incidents suggested any truth in the circulating rumours. This situation also illustrates the complex and constantly shifting relation between and within research and community groups.

Discussions around what was expected at the end of the study re-emphasised the importance of inter-personal relations and trust, and the fragility of both. While there was a clear interest in finding out the trial outcome, there was also a desire to prove rumour-mongers wrong, and to find out what would happen if ‘KEMRI's children’ suffered future problems. Most evident here were the intra-community tensions, jealousies and feelings of superiority that were woven into those interests. Thus reasons given for needing feedback, and particularly positive feedback, included making non-participants regret their decision not to be involved and to absolve those who had aligned themselves with the study team. The latter was often described as a major commitment and show of trust given the repeated efforts from some community members to undermine their decision. Occasionally, the local FW – seen as a key connection point in the alliance between participants and KEMRI staff, and generally praised for his/her general assistance in the vaccine trial – was threatened when the possibility of negative findings were raised by group discussion participants:

“… let us pray that the year ends without any bad incident and new year starts nicely but in case one child dies who is in their group… [FW threatened] (M2; p 37).

There was clearly a concern that the benefits and privileged status of participants vis-à-vis non-participants should not be ended suddenly, particularly the regular surveillance of children's health. As was described in one group, if you do so “we'll become a laughing stock” (M2; P12; p 32). Such comments show how community members can contribute to debates on locally appropriate ethical practice, including around appropriate levels of benefits for participation and what to do when a trial is over.

The above findings suggest that participation in research can contribute to the forming of new types of social relationships or even new ‘communities’ (i.e., ‘groups of people with diverse characteristics who are linked by social ties, share common perspectives and engage in joint action in geographical locations or settings’ (MacQueen et al., 2001, p 1936). Fieldworkers based ‘in the field’ can also play an important role in the establishment, maintenance and nature of these communities, and can contribute to and be impacted on by relationships between these communities. Exploring these issues, and more specifically the meaning of membership in communities of ‘trial participants’ and ‘non-participants’, their relative importance over other social networks and over time, tensions and shifts between them, and how membership is sought, maintained and ended in each, was beyond the scope of our study. Such information would require more individual in-depth interviews and ideally a more thorough immersion and residence in the community itself. The data would be invaluable not only to informing information-giving processes, but also to better understanding the experiences and consequences – and, therefore, ethical implications – of research activities.

## Conclusions

Our findings illustrate that inter-personal interactions and relationships between researchers and community members, and within the community, are critical to participants' perceptions of and decisions to join or leave a study. Responsive community engagement activities, including careful informed consent procedures, can help identify and respond to emerging issues, and build trusting relationships that are supportive of positive perceptions and informed participation. However, these relationships are also based on and continually tested by concerns and interests that can be difficult to predict, or that are well beyond the timescale and reach of single research activities. Fieldworkers at the interface between research groups and communities have a particularly key and often difficult role in establishing and maintaining these interactions and relationships. Their critical role in ethical practice at the field level through answering community members' own practical and ethical comments, raised through, for example, questions, concerns and rumours, is often under-recognised and under-supported.

We have highlighted in our discussion some areas that would benefit from further in-depth exploration, and from comparison in other settings and by other study types. Nevertheless, we would counter-balance the current move towards increasingly ambitious and stringent formal standards for individual information-giving and checking, with a call for greater attention to the social relationships that are clearly affected by and essential to the success of these procedures. In so doing, we support others (Weijer & Emanuel, 2000; Weijer, Goldsand, & Emanuel, 1999) in emphasising respect for communities as well as persons, and in recognising that current guidelines and regulations are an inadequate response to the complex, often unpredictable and ever shifting ethical dilemmas facing researchers ‘in the field’ (Mitchell et al., 2002). In Kilifi, we are strengthening our efforts to respond to these issues through the development and implementation of a communication strategy, including community engagement activities. These initiatives, and the dilemmas and lessons we have learned to date, are described in detail elsewhere (Marsh et al., 2008). These papers not only offer practical ideas that we believe have been valuable in our setting, but also illustrate that striving for improved ethical practice will always be challenging, incomplete, and involve complex and open processes of discussion, interaction and negotiation between parties with diverse interests.

## Figures and Tables

**Table 1:** Correct and don't know answers on key vaccine trial information (quiz)

Questions	Correct response (%)	Stating DK (%)
What is this research for?	35	26
Why vaccine can't be given to all Kenyan children at the moment?	48	45
If your child is healthy, and take part, how many injections will they receive?	49	31
Will all children be given a malaria vaccine?	85	16
Why will you have to stay at the clinic for 1 h after the injection?	80	14
Will children who have received a vaccine be able to get malaria?	29	44

## Box 1 Summary of MVT procedures

• Preliminary health check/screening at the local dispensary, including blood-taking, urine and stool sampling, height and weight measurements, and a physical check. A photograph for identification on subsequent visits.
• For children whose parents consented, and who were well enough to participate, vaccine administration in three injections over three subsequent visits to the dispensary. Children stay for 1 h after each vaccination to observe for any immediate side-effects.
• Daily check-ups for 3 days, and one week, after each vaccination.
• After the last vaccination, all children treated for malaria, and a blood test taken.
• Weekly home visits to monitor for malaria for one year, including blood sample collection after 3 months and at one year.

## Box 2 Summary of MVT information-giving processes

• PI and his team met with local dispensary committee and local administration to seek permission to conduct the study in the area and to discuss the study details and practicalities.
• A series of large community sensitisation meetings in each of the specific villages. These were public meetings already organised by local leaders, attended by the PI and fieldworkers. There was an oral presentation of the study aims and design, and information sheets were distributed. Questioning of the PI and FWs by the community and community representatives was encouraged.
• MVT fieldworkers made individual visits to homesteads to discuss the study in more detail and to invite parents to bring their children to the local dispensary for further information and, should they choose to consent, for screening.
• At the dispensary, the MVT information was repeated to parents in groups as they arrived, and then to individual parents before they signed consent and the screening/health check was done. Time was given for questioning and discussion at both the group and individual stage.
• A further, brief explanation was given prior to administration of the vaccine, which occurred at least one week after giving consent.

## Box 3 Reported bases of ‘devil worshipping’ rumours

• Blood sampling: are volumes taken not dangerous? what is done with it? where does it go?
• Fear of whites: Why are they here? Have they returned to re-colonise us? They have ‘their own complexities’ ‘they're not trusted’, ‘they have jinis (demons)’
• Distribution of free things: ‘we've always had health problems why come now? what can their motivation be? why give sodas, juices, bread? why visit people in their homes even at midnight?’
• Other screening procedures: are the measurements of a child's height and head circumference taken as part of the initial health check actually measurements for the child's coffin? Can photographs be taken away and used to cause harm to the child from elsewhere?
• A failure or refusal by rumour-mongers to listen to or internalise study information; and
• Ultimately, a fear that children will later lose their good health, get paralysed, fit or die.

## Box 4 Examples of FWs responses to parents' concerns about devil worship

I would explain that the blood from your child alone cannot be enough for devil worshippers because devil worshippers would need an amount maybe a glass size or more… [P2; page 6].
You know in my area there were few people with such rumours and I tried to explain to them that KEMRI is an organisation which was started lawfully by the Kenyan parliament so this is not a thing that the government doesn't know and also…the local leaders have been consulted [goes on to explain the historical reasons for the logo which was apparently fuelling rumours] [P10; page 8].

## Footnotes

1 This was a multiple response question.
